# Long-term monitoring data of hygrothermal conditions of a retrofitted historic building in Settequerce, South Tyrol (Italy)

**DOI:** 10.1016/j.dib.2024.110137

**Published:** 2024-02-01

**Authors:** Simone Panico, Marco Larcher, Daniel Herrera-Avellanosa, David Cennamo, Alexandra Troi

**Affiliations:** Institute for Renewable Energies, Eurac Research, Viale Druso 1, 39100 Bolzano, Italy

**Keywords:** Historic building, Retrofitting, Hygrothermal conditions, Data monitoring

## Abstract

This data collection presents a comprehensive five-year monitoring record from a historic building located in Settequerce, South Tyrol (Italy). The 17th-century building, characterized by a stone masonry, was subject to a retrofit in 2017 that introduced a wood fiber insulation panel to the inner side of the building's walls. As part of the renovation process, a monitoring system was installed. This system incorporated internal, external, and in-wall sensors, which were strategically placed at three different points within the wall to evaluate the effects of the insulation on the stratigraphy. This setup ensured the continuous collection of climatic data, both from the exterior and interior environments, as well as from within the renovated wall's stratigraphy. This comprehensive dataset delivers new insights into the long-term performance of retrofit interventions. The significant potential for reuse of this dataset makes it a substantial foundation for future studies on energy-efficient retrofitting and the preservation of historical structures in comparable climate (Climate E Köppen). It is expected to inform data-driven decisions in future renovation and preservation projects of comparable historic buildings.

Specifications Table

**Table:** 

Subject	Civil and Structural Engineering
Specific subject area	Monitoring of the hygrothermal conditions of an interior insulated historic wall and its surrounding indoor and outdoor climate conditions.
Type of data	Table Graph
How the data were acquired	Data were collected using a comprehensive monitoring system including external, internal, and in-wall sensors. The hardware used consists of *E* + *E* EE060 temperature and relative humidity sensors and a Hukseflux SR05 pyranometer. Data were logged via a wired control unit. The monitoring system captured climatic conditions over a span of five years, providing continuous measurements both inside and outside the building, as well as within the retrofitted wall at three different depths. Further details on data collection methodologies are elucidated in references [1], [2], [3], [4], [5].
Data format	Raw Analyzed
Description of data collection	Data were collected every five minutes from varying conditions within and around a retrofitted historic building over a span of five years. Post-processing was conducted to derive hourly averages. Occasional data gaps due to sensor malfunction were noted. No exclusion criteria were applied, and all recorded data were included for comprehensive analysis.
Data source location	· Institution: Eurac Research · City/Town/Region: Settequerce, South Tyrol · Country: Italy · Latitude and Longitude: 46.5085° N, 11.2646° E
Data accessibility	Panico, Simone (2023), “Comprehensive Five-Year Dataset: Internal, External and In-Wall Climate Monitoring of a Retrofitted Historic Building in South Tyrol”, Mendeley Data, V1, doi: 10.17632/s8mnkgkmbs.1 Direct URL to data: https://data.mendeley.com/datasets/s8mnkgkmbs/1
Related research article	[4] S. Panico, M. Larcher, V. Marincioni, A. Troi, C. Baglivo, P.M. Congedo, Identifying key parameters through a sensitivity analysis for realistic hygrothermal simulations at wall level supported by monitored data, Build Environ. 229 (2023) 109969. https://doi.org/10.1016/J.BUILDENV.2022.109969.

## 1 Value of the Data

• These data provide valuable insights into the long-term thermal and hygric behavior of internally insulated historic masonry walls. This is especially relevant for those located in regions with similar climatic conditions to Northern Italy. Researchers and professionals in the field of building preservation, civil and structural engineering, and energy efficiency can benefit from these data. Analyses and findings derived from these data can be informative for policymakers and organizations focusing on historic preservation and energy retrofitting.
• The data set can be used to validate and calibrate numerical models, with the aim of increasing their accuracy and reliability. This dataset is especially crucial given the increasing focus on sustainable renovation practices for historic buildings, which are often challenging to preserve while improving energy efficiency.
• In addition, the data can be used to reduce the skepticism of designers with regard to internal insulation systems and thus encourage the diffusion of this type of solution to increase the energy efficiency of historic buildings.
• The climatic data can be used to study the effects of local climate on the performance of retrofitted masonry walls, potentially serving as a benchmark for studies focusing on climate effects on retrofitted structures.

## 2 Objective

This dataset was generated as part of an extensive monitoring project aimed at investigating the long-term thermal and hygric behavior of a retrofitted historic building in Northern Italy.

The monitoring system was designed to collect data not only on external and internal climatic conditions but also within the wall stratigraphy at different layers, offering a comprehensive understanding of the wall's performance. Given the dearth of long-term performance data for retrofitted historic buildings in similar climatic zones, this dataset fills a significant gap. It provides a unique empirical foundation for refining numerical models, evaluating retrofit measures, and understanding the impact of local climate on building performance, making it an invaluable resource for future research and practice. Moreover, the objective of capturing this dataset aligns with the global initiative to enhance the sustainability of built heritage, providing empirical data to support evidence-based conservation strategies.

## 3 Data Description

**Fig. 1:**
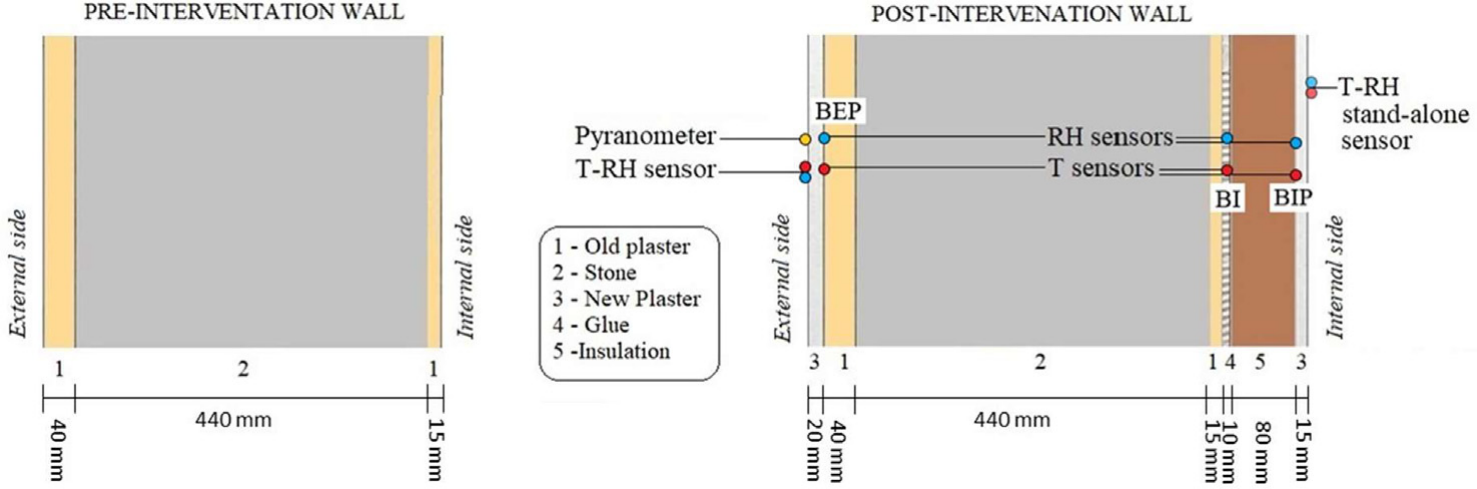
Representation of the stratigraphy of the wall under investigation before and after renovation.

The building, dating back to the 17th century, underwent a retrofit intervention in 2017 which included the addition of a wood fiber insulation panel to the interior side of its stone walls. Fig. 1 illustrates the stratigraphy's materials before and after the renovation. It also shows type and position of the sensors which will be described in the next sections.

**Fig. 2:**
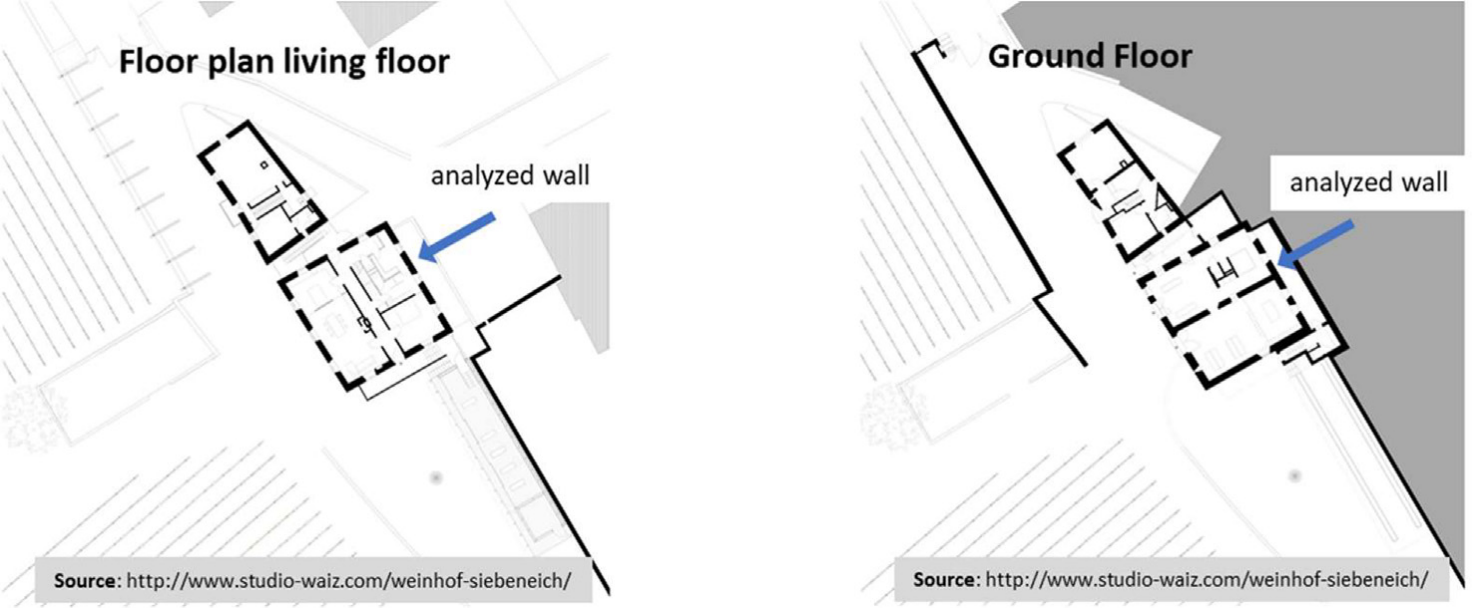
Ground and living floor planimetry. The monitored zone is marked with a blue arrow.

The detailed insights into the thermal and hygric performance gained from this study contribute to the growing body of research on climate adaptability and resilience of historic structures. To complement the data, the included floor plans (refer to Fig. 2) indicate the monitored wall's location with a blue arrow, providing context for the sensor data to be discussed in subsequent sections.

The raw data files available in the repository (**Settequerce_Case_Study.csv**) represent values read by the monitoring system's sensors. The measurements range from January 19, 2018, at 10:00:00 to February 27, 2023, at 15:10:20, spanning a total of 5 years and 1 month. These measurements offer detailed climatic information collected from multiple locations: externally, internally, and within three different layers of the wall stratigraphy. Within each layer, two sensors were placed to measure temperature (T) and relative humidity (RH), respectively labeled as 1 and 2. The naming convention used for the datasets is as follows:

Their high-resolution and long-term nature, combined with the range of positions, provide an opportunity to understand the building's thermal and hygric performance over an extended period.

It's important to note that the monitoring system, while robust, experienced occasional malfunctions that resulted in missing data for certain periods. Particularly, the internal sensor, which operates as a stand-alone unit, faced separate periods of data recording issues. In addition to the data gaps listed in Table 1 with a duration greater than 5 days, there were other shorter gaps. Specifically, for the monitoring system, there were 3 data gaps in 2018, 4 in 2019, 3 in 2020, 2 in 2021, 23 in 2022, and 2 in 2023. For the stand-alone internal sensor, there was an additional gap of 4 h in 2021 beyond what is listed in the table. These potential gaps in data should be considered when examining the datasets. Table 1 shows the gap time ranges for each sensor in the monitoring system and the internal stand-alone sensor (only gaps greater than 5 days are reported) (Tables 2 and 3).

**Table 1:** Nomenclature used to indicate where the sensors are placed.

Position	Description
Ext	Sensors placed on the External side
Int	Sensors placed on the Internal side
BI	Sensors placed Behind the Insulation layer
BIP	Sensors placed Behind the Internal Plaster layer
BEP	Sensors placed Behind the External Plaster layer

**Table 2:** Nomenclature used to indicate the type of data recorded by the sensor.

Measure Type	Description
T	Temperature sensors
RH	Relative humidity sensors
Solar_Radiation	Pyranometer sensor data

**Table 3:** Time range of gaps for each sensor of the monitoring system and internal stand-alone sensor.

Data Gaps	start	end	duration
**Monitoring System - All Sensors**	25.03.21 10:00	16.12.21 14:00	266 days 04 h
	16.05.22 15:00	23.05.22 07:00	6 days 16 h
	08.06.22 16:00	14.06.22 07:00	5 days 15 h
	04.07.22 01:00	13.07.22 20:00	9 days 19 h
	26.07.22 01:00	02.08.22 10:00	7 days 09 h
	10.08.22 01:00	19.08.22 22:00	9 days 21 h
	22.08.22 18:00	08.09.22 11:00	16 days 17 h
	16.09.22 04:00	30.09.22 23:00	14 days 19 h
**Stand-alone Internal Sensor**	30.12.19 19:00	11.12.20 11:00	346 days 16 h

Furthermore, some inspections revealed an increase in vegetation in the direction of the pyranometer. This goes hand in hand with a reduction in the data values recorded by the pyranometer over the years.

**Fig. 3:**
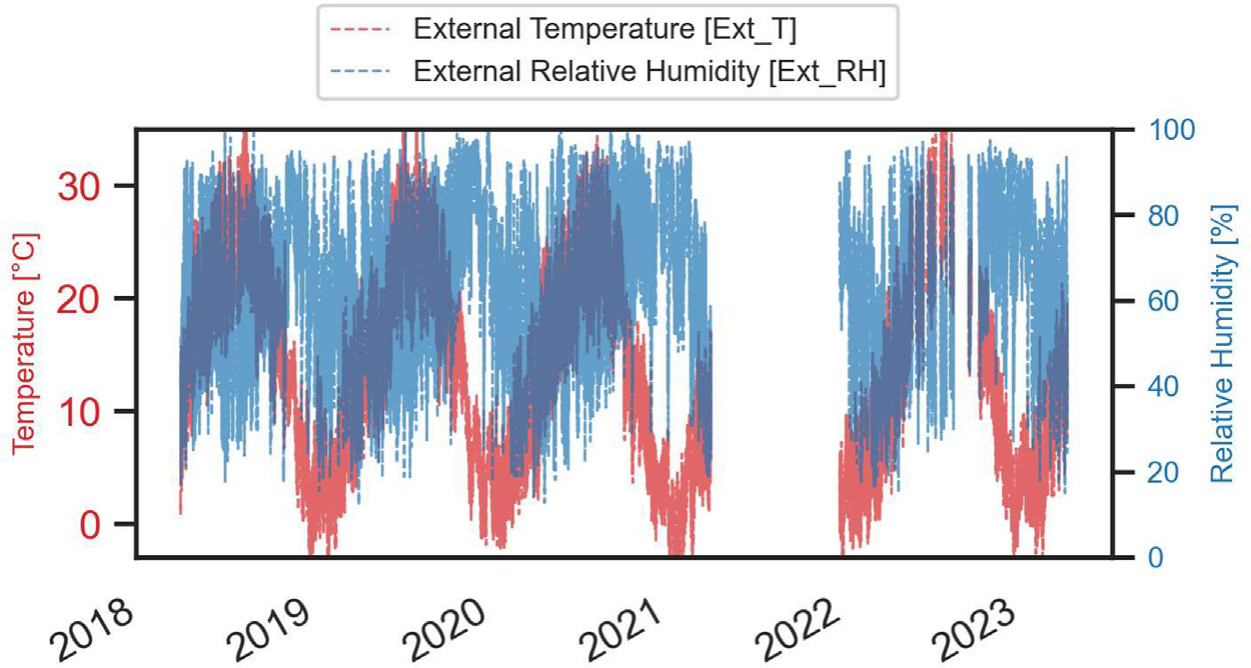
Temperature and relative humidity values recorded by the external sensor placed near the outer wall.

**Fig. 4:**
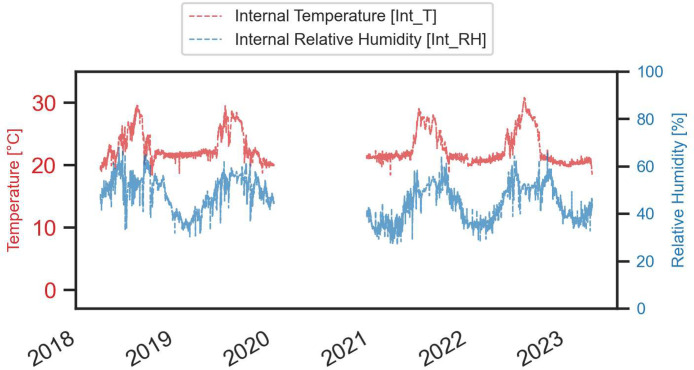
Internal temperature and relative humidity values recorded by the stand-alone sensor placed inside the building.

**Fig. 5:**
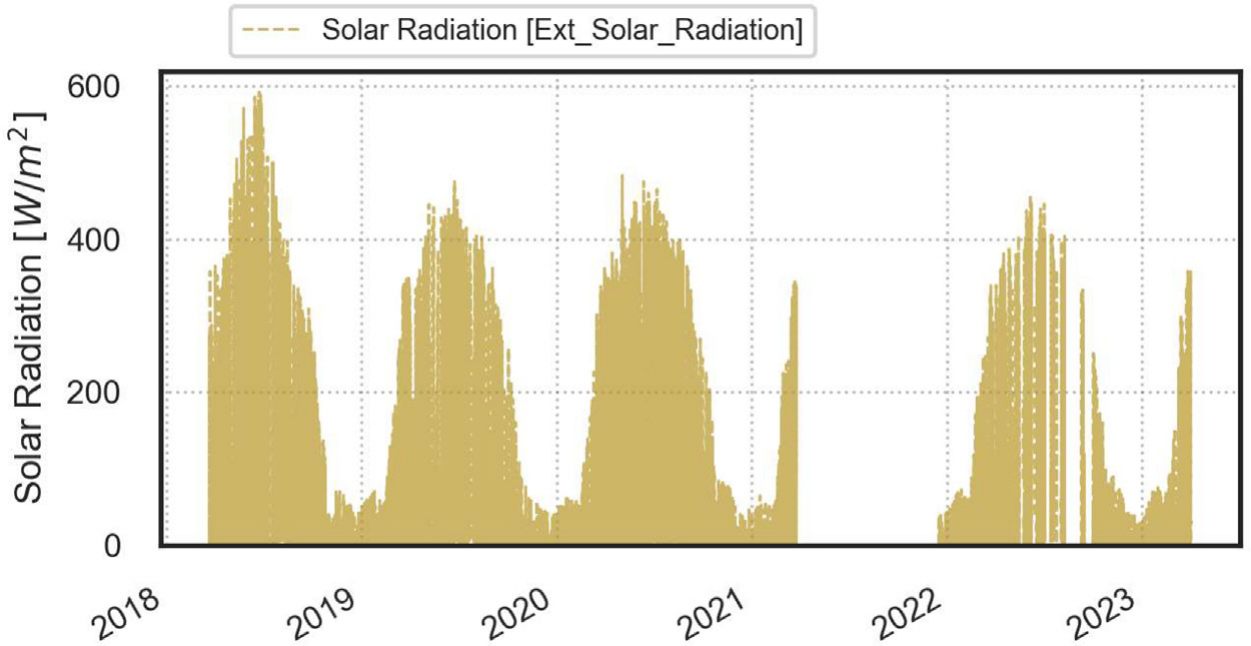
Solar radiation values recorded by the pyranometer positioned on the outer wall.

**Fig. 6:**
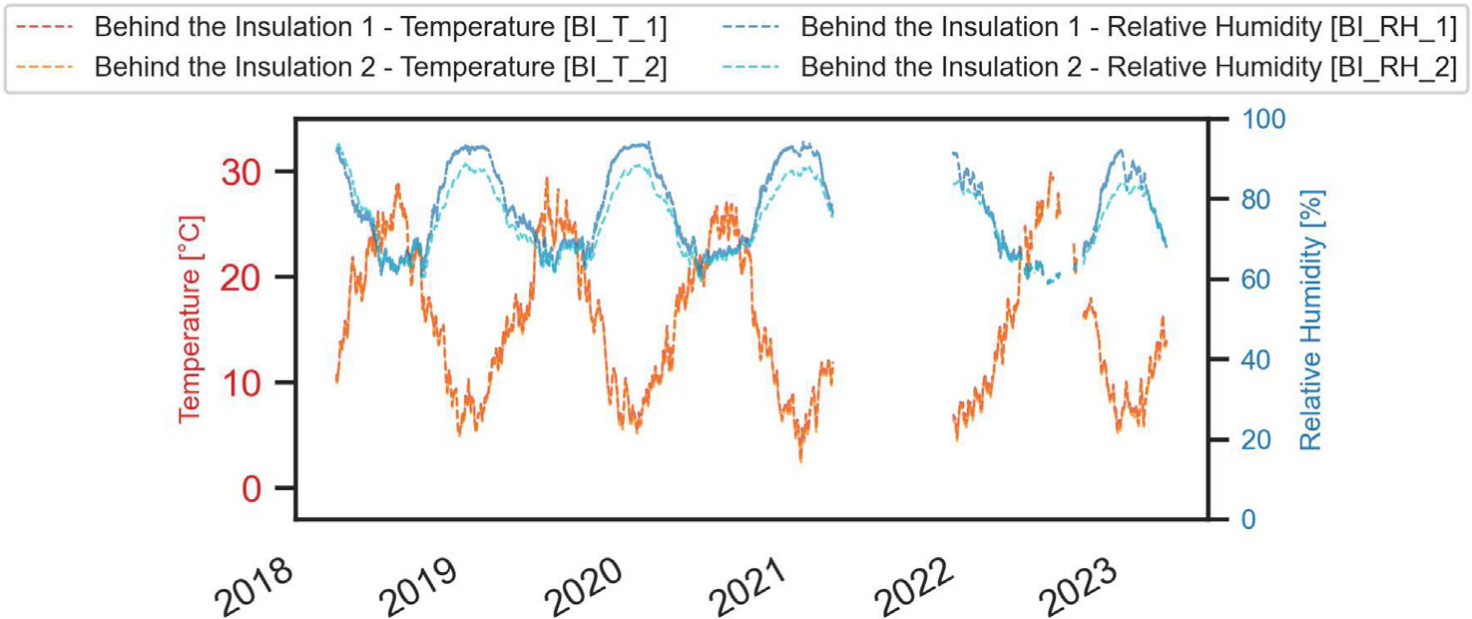
Temperature and relative humidity values recorded by the two sensors positioned in the layer between the insulation and the historic plaster.

**Fig. 7:**
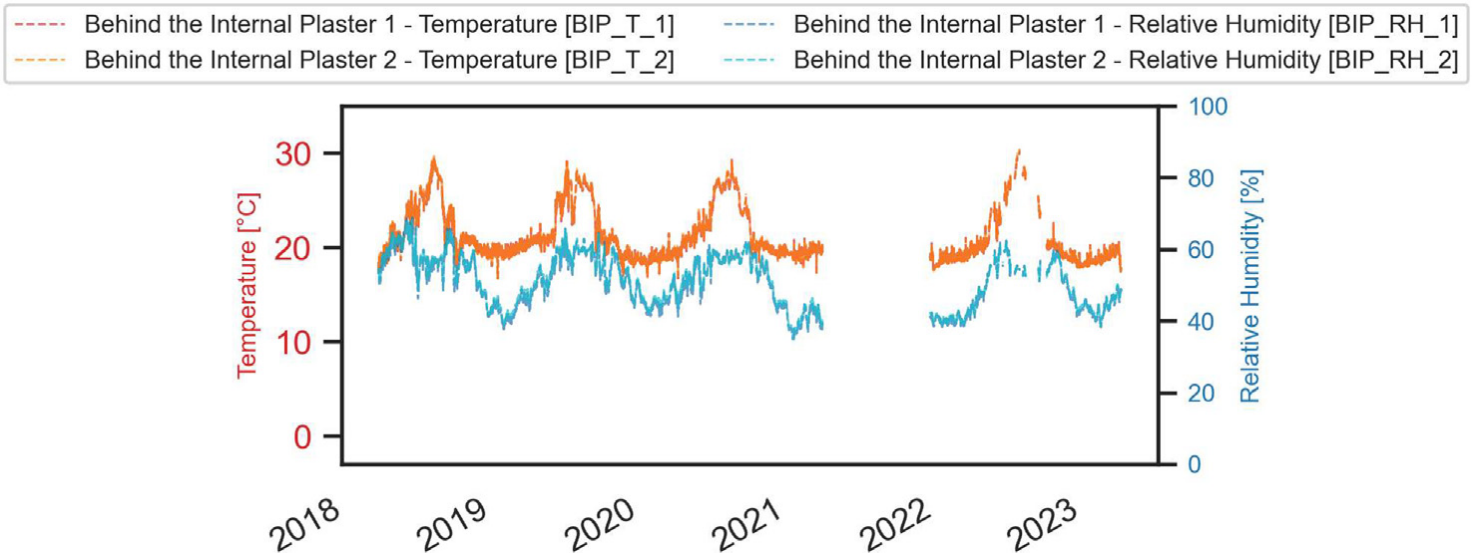
Temperature and relative humidity values recorded by the two sensors positioned beneath the internal plaster (on the insulation from the internal side).

**Fig. 8:**
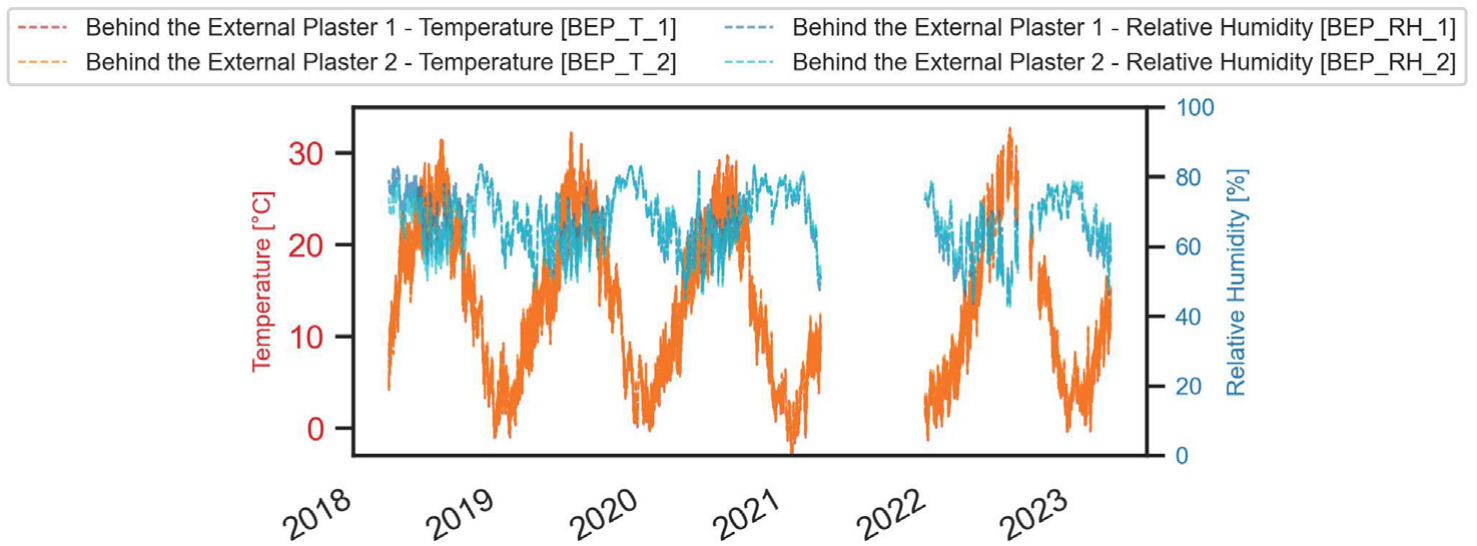
Temperature and relative humidity values recorded under the external plaster.

A comprehensive visual representation of the monitored data is provided in the next figures (Fig. 3, Fig. 4, Fig. 5, Fig. 6, Fig. 7, Fig. 8).

## 4 Experimental Design, Materials and Methods

The study at hand presents a case involving a historic residential building located in Settequerce, South Tyrol (Italy). This building falls under the Italian climatic classification zone E, characterized by 2771 heating degree days. For the international classification proposed by Köppen, the building falls into the Dfb climate class, which is defined as a humid continental climate with a warm summer.

The primary focus of this study lies in examining the hygrothermal behavior of the internally insulated building walls, focusing, in particular, on the one which faces Northeast (azimuth of 62° from the North). The area delineated by the monitored wall serves as a storage room for the owners but is also frequently used as a laundry room.

The monitored wall is a composite of existing (historical) materials and new ones installed during a recent energy retrofit intervention in 2017. Prior to the refurbishment, the existing wall configuration consisted of 4 cm external plaster, 44 cm red porphyry masonry, and 1.5 cm internal plaster.

During the building's energy retrofitting, an 8 cm wooden fiber panel was added to the inner side, glued to the old plaster with a clay-based adhesive approximately 1 cm thick. Furthermore, the renovation phase included the application of lime-based plaster on both the internal (1.5 cm) and external facades (2 cm).

The monitoring system was designed to record the exterior climate of the location, the interior building climate, and the hygrothermal conditions within the wall. The exterior climate is monitored using temperature and relative humidity sensors (*E* + *E* EE060) shielded against solar radiation and a pyranometer (Hukseflux SR05) for the acquisition of incident solar radiation, installed directly on the wall.

A stand-alone temperature and relative humidity sensor (*E* + *E* HUMLOG 20) is situated in the interior room, providing all the necessary information about the indoor climate.

To monitor the conditions within the wall, combined temperature and relative humidity sensors were installed at the interfaces between the different materials. The installation of these sensors occurred during the renovation phase; thus, it was possible to avoid drilling holes in the wall, preventing the formation of thermal bridges that could affect the reliability of the recorded values.

These sensors were connected to a suitably designed monitoring system to save the data. It consisted of Seneca cards connected to the sensors for analog data acquisition to be transmitted via modbus-rtu, a 24 VDC–50 W power supply unit, an industrial computer equipped with Windows 10 connected to a data acquisition and to a gsm router. This router, equipped with an internal sim card, permits internet connectivity to enable remote data download and system maintenance.

The acquisition software was developed using Labview to acquire and record data using a configurable acquisition time. Data are recorded at a configurable storage time using a csv file on daily basis, in this case 5 min are used. The data were subsequently read with a Python script, through which hourly averages were computed.

## Ethics Statements

This work does not contain any studies with human or animal subjects.

## CRediT authorship contribution statement

**Simone Panico:** Software, Formal analysis, Investigation, Data curation, Writing – original draft, Visualization. **Marco Larcher:** Conceptualization, Methodology, Investigation, Project administration. **Daniel Herrera-Avellanosa:** Validation, Writing – review & editing, Supervision, Project administration. **David Cennamo:** Writing – review & editing. **Alexandra Troi:** Methodology, Validation, Writing – review & editing, Funding acquisition.

## Declaration of Competing Interest

The authors declare that they have no known competing financial interests or personal relationships that could have appeared to influence the work reported in this paper.

## Data Availability

Comprehensive Five-Year Dataset: Internal, External and In-Wall Climate Monitoring of a Retrofitted Historic Building in South Tyrol (Original data) (Mendeley Data).
